# Social redistribution of pain and money

**DOI:** 10.1038/srep15389

**Published:** 2015-10-30

**Authors:** Giles W. Story, Ivo Vlaev, Robert D. Metcalfe, Molly J. Crockett, Zeb Kurth-Nelson, Ara Darzi, Raymond J. Dolan

**Affiliations:** 1 Centre for Health Policy, Institute of Global Health Innovation, Imperial College London, London, 10^th^ Floor, St. Mary’s Hospital, London, W2 1NY, UK; 2 Wellcome Trust Centre for Neuroimaging, University College London, London, WC1N 3BG UK; 3 Warwick Business School, The University of Warwick, Coventry, CV4 7AL, UK; 4 Becker Friedman Institute, University of Chicago, 575 S. University Ave., Chicago, IL 60637, US; 5 Max Planck UCL Centre for Computational Psychiatry and Ageing Research, UCL, c/o Wellcome Trust Centre for Neuroimaging, University College London, London, WC1N 3BG UK; 6 Department of Experimental Psychology, University of Oxford, 9 South Parks Road, Oxford OX1 3UD, UK

## Abstract

People show empathic responses to others’ pain, yet how they choose to apportion pain between themselves and others is not well understood. To address this question, we observed choices to reapportion social allocations of painful stimuli and, for comparison, also elicited equivalent choices with money. On average people sought to equalize allocations of both pain and money, in a manner which indicated that inequality carried an increasing marginal cost. Preferences for pain were more altruistic than for money, with several participants assigning more than half the pain to themselves. Our data indicate that, given concern for others, the fundamental principle of diminishing marginal utility motivates spreading costs across individuals. A model incorporating this assumption outperformed existing models of social utility in explaining the data. By implementing selected allocations for real, we also found that while inequality *per se* did not influence pain perception, altruistic behavior had an intrinsic analgesic effect for the recipient.

Social inequality is a near universal feature of human experience. Nevertheless, people often appear motivated to reduce inequality, for example increased wealth drives charitable action^1^,^2^, and relative poverty is associated with acquisitive crime^3^. A teleological explanation for such behavior is that people are concerned with achieving a fair (equitable) social distribution of benefits^4^. The latter is often approximated as a subjective cost associated with having either more or fewer resources than others, termed inequality aversion^5^,^6^,^7^,^8^,^9^.

Many experimental studies have examined how humans redress monetary inequality. A common observation is that within a scenario referred to as the ‘dictator game’, people endowed with money frequently share a portion with others in the absence of direct return for themselves^2^,^4^,^10^,^11^. Similarly people will take money from those endowed with more money than themselves^10^,^12^. By contrast, how humans respond to unequal allocations of *pain* between themselves and others is little studied.

Commensurate with documented empathic responses to pain in others^13^,^14^,^15^, participants take on painful stimuli to relieve the apparent suffering of a confederate^16^,^17^. Furthermore, a study implementing a dictator game with painful outcomes found that participants were particularly egalitarian, allocating on average 48% of the pain (time spent immersing one’s hand in ice water) to themselves, while in a monetary dictator game the same participants were significantly less charitable, donating only 30% of the endowment on average^18^. These findings might suggest that people are more inequality-averse for pain than money, in other words that people particularly dislike having either more or less pain than others. However an alternative possibility is that people are simply more altruistic for pain than for money, in the sense that relieving others’ of their physical suffering carries greater motivational weight than does increasing others’ wealth.

In support of the idea that pain evokes particularly altruistic responses, a recent study found that participants demanded higher monetary compensation in return for an increase in another participant’s pain than for an increase in their own pain^19^. The latter behavior, referred to as ‘hyperaltruism’, was taken to imply that participants in fact assigned greater disvalue to harming others than to harming themselves. In a pain dictator game, hyperaltruistic participants ought to take on more pain than they allocate the other player; indeed in the dictator game study described above, 30% of participants retained more than half the pain, while only 2% gave away more than half the money^18^.

A key question emerges, namely how can a putative preference for equal allocations of pain be reconciled with hyperaltruism? A key aim of the current study was to address this question by comparing the ability of alternative social utility models to account for behavior in a modified pain dictator game. Importantly, models of social utility which solely implement an heuristic penalty for inequality^5^,^6^ cannot account for hyperaltruism. These models assume that people derive utility from the size of their own payoff, but incur a utility cost to having either more or fewer resources than others. Under the simplest of such models^6^, both sources of utility are assumed to be linear. Such a model predicts that in dictator games with an endowment of reward people should either retain all an endowment for themselves (offering nothing to the other player) or split the endowment equally. This model cannot account for the finding that in dictator games many people choose other allocations that are neither maximally selfish nor maximally equal ones^10^, of which hyperaltruistic allocations for pain are an example^18^. A more nuanced account implements a non-linear penalty for inequality^5^. This model allows for a preference in dictator games to offer the other player something less than half of the endowment, but is nevertheless unable to account for hyperaltruistic offers. Here we show that both hyperaltruism and equality-seeking can be accounted for within a social utility model in which the utility of one’s own and others’ payoffs are combined in a weighted sum^20^, but where each are non-linear in the size of the payoff (obey diminishing marginal utility for rewards and increasing marginal disutility for pain). Under this model, given that people have some degree of concern for others, inequality aversion derives from the more fundamental principle of diminishing marginal utility over individual payoffs.

The conventional dictator game setup is not ideally suited to disambiguating alternative models of social utility, since it provides information only about a person’s preferred allocation, and does not allow for an estimate of how a person values different degrees of pain inequality (pertinent for example to whether inequality carries a linear or non-linear cost). To address this, we implemented a modified dictator game with mildly painful cutaneous electric shocks as the outcomes, where participants were given an opportunity to leave unchanged or adjust default allocations of 24 shocks between themselves and an anonymous recipient. Adjustments could be made by giving or taking fixed numbers of shocks. Three ‘action frames’ were implemented: a *Give* frame, a *Take* frame and a *Give or Take* frame, with names corresponding to the permissible actions (Fig. 1). By observing how frequently participants adjusted the default allocations at differing levels of inequality, we could map a pattern of inequality aversion for pain, albeit coarsely.

We also aimed to test an hypothesis, supported by previous studies, that people are more altruistic when allocating pain, compared with money. To do so, in a subset of participants, we implemented an equivalent set of choices with monetary outcomes where, in two sub-conditions, these choices were framed as either gains or losses of money, where losses were implemented as debts to be repaid from an endowment. We compared allocations of monetary losses (debt) with those of pain, since these conditions were matched for valence. We predicted that participants would adjust the default allocations in the direction of equality for both pain and debt; based on greater altruism for pain than money in standard dictator games^18^, we predicted that participants would allocate more debt than pain to the other participant. Finally, based on existing findings of hyperaltruism for pain^18^,^19^ we also predicted that hyperaltruism would be more prevalent for pain than for debt.

In addition to the above, as it is known that social comparison can influence responses to reward^9^,^21^,^22^, we were interested in whether and how pain inequality influences pain perception. In the real world perceived pain inequality is likely to result from subjective judgments of pain. Therefore, if disadvantageous inequality were to increase pain perception, this might engender positive feedback, whereby disadvantageous inequality increases subjective pain, which in turn increases perceived pain inequality, and so on. Such feedback might contribute, for example, to the maintenance of chronic pain syndromes. Providing indirect support for this idea, amongst sufferers of chronic pain, perceived injustice is known to be associated with greater subjective pain and disability^23^,^24^. We therefore tested the hypothesis that advantageous inequality reduces pain perception, while disadvantageous inequality increases it. Based also on existing findings showing that social rejection and physical pain have overlapping representations^23^,^24^,^25^,^26^,^27^,^28^,^29^ a further prediction is that pain resulting from the benevolent actions of another person is perceived as less severe than an equivalent unintentional pain, and *vice versa*. Two previous studies support this prediction, though these studies did not directly test an effect of inequality^30^,^31^.

To test the above hypotheses, we implemented selected allocations of pain for real, attributing them either to the play of chance or to the decisions of the dictator (shown in Fig. 2), and then asked subjects to rate the intensity of the painful stimuli. Since this experiment was performed in the same participants as took part in the pain dictator game, and since outcomes of the dictator game were restricted to a discrete set of possible allocations, we could inform participants in good faith that the experienced allocations attributed to the decisions of the other player had indeed been chosen by the other player (albeit not necessarily in the same proportions as presented, a fact which participants were also informed of).

## Results

### Dictator Behavior

#### Distribution of Mean Offers

The distribution of offers across participants (taking the mean resulting offer over all three action frames for each participant) is displayed in Fig. 3 for pain (Fig. 3a) and money (Fig. 3b for losses, 3c for gains). Across participants the mean offer of shocks was not significantly different from 12 shocks (*N* = 47; mean = 12.16, permutation test, p > 0.25, see Methods), indicating a preference for equal allocations of pain at the group level. By contrast the mean offer of money (averaged across gain and loss frames) was significantly below £12 (*N* = 25; mean = £11.39, p = 0.011, two-tailed permutation test), indicating a degree of self-oriented behavior for monetary outcomes.

To compare the degree of self-oriented behavior for money and shocks we focused on comparing offers in pain and money loss conditions, since these were matched for valence. As predicted, offers of shocks were significantly lower than offers of debt (mean shocks offered in 25 subjects performing money choices = 11.98, mean debt offered = £12.98; p = 0.021, two-tailed permutation test, uncorrected; p = 0.042 Bonferroni corrected for two possible comparisons, pain vs money gain or pain vs money loss). Surprisingly there was no significant difference between the mean offers in pain and money gain conditions (mean money retained by dictator = £12.24; p > 0.250), nor was there a difference between money gain and money loss conditions (p > 0.250).

We note that, given the restricted nature of the choices, this analysis based on mean offers might not be the most sensitive for detecting changes in altruism between modalities. Nevertheless, taken together these results indicate that participants were most altruistic when apportioning pain, and least altruistic when apportioning debt. We perform additional analysis on the raw choice frequencies below, by comparing responses to advantageous and disadvantageous inequality for pain and debt.

We tested for hyperaltruism by examining the frequency with which dictators gave away less than half of the shocks, or more than half of the money. For pain allocations, 15 out of 47 participants (32%) retained more than half the shocks across all choices, indicating hyperaltruistic behavior. By contrast, in the money loss frame, only 3 out of 25 participants (12%) assigned more than half of the money to the receiver (one-sided Fisher’s exact test p = 0.054).

### Inequality Aversion for Pain and Money

To examine the pattern of social preferences in more detail, we analyzed the frequency with which participants altered the default allocations as inequality changed. In the *Take* frame participants were increasingly likely to take a fixed number of shocks from the other participant as the default number of shocks allocated to the other participant increased (Fig. 4a). Similarly in the *Give* frame, participants became less likely to give shocks to the other participant as the baseline number of shocks allocated to the other participant increased (Fig. 4b). An equivalent effect was seen in the *Give or Take* frame (Fig. 4c,d). In the monetary domain a similar effect was also observed (Fig. 5). Importantly, this particular pattern is in keeping with an increasing marginal effect of inequality, since the propensity to correct inequality by the same degree, 6 shocks, increased as the baseline level of inequality increased (from 6–18 to 0–24 in the Take frame, and from 18–6 to 24–0 in the Give frame).

### Self-oriented Preferences for Monetary Losses but not Pain

To further compare social preferences for debt and pain at the group level, we separated choices (in the *Give* and *Take* action frames) which entailed disadvantageous inequality (24–0 vs 18–6, 18–6 vs 12–12) from those which entailed advantageous inequality (6–18 vs 0–24, 12–12 vs 6–18). Here, choosing the more equal option in ‘disadvantageous’ choices more frequently than in the symmetrical ‘advantageous’ choices indicates a degree of self-oriented behavior. This analysis creates a three-way factorial design with two outcome modalities (debt vs pain), two action frames (*Give* vs *Take*) and two directions of inequality (advantageous vs disadvantageous). A main effect of modality would indicate greater or lesser inequality aversion for pain compared with debt irrespective of the direction of inequality. By contrast a modality x inequality-direction interaction would indicate more or less altruistic behavior for pain.

Three-way repeated measures ANOVA on the probability of choosing the more equal option (termed equality-seeking) for the 25 participants who made choices for both modalities revealed a significant main effect of inequality-direction (*F*(1,24) = 4.616, p = 0.042), driven by some degree of self-oriented behavior irrespective of modality or action frame (estimated marginal mean equality-seeking = 0.745 for advantageous choices, 0.872 for disadvantageous choices). There was neither a significant main effect of modality (*F*(1,24) = 0.183, p > 0.250), nor a significant main effect of action (*F*(1,24) = 2.348, p = 0.139). Importantly, as predicted, there was a significant interaction between modality and inequality-direction (*F*(1,24) = 6.233, p = 0.020). Follow-up non-parametric tests (by randomly reshuffling condition assignment) indicated that this was driven by greater self-oriented behavior for debt than for pain. When allocating debt participants were on average 26% more likely to choose the more equal option in disadvantageous choices than in advantageous choices (mean difference = 0.26, two-tailed p < 0.001, *N* = 25). By contrast, for pain there was no significant difference in equality-seeking between disadvantageous and advantageous choices (mean difference <0.01, two-tailed p > 0.250, *N* = 25). Furthermore, the observed asymmetry for debt was significantly greater than that for pain (two-tailed p = 0.018, *N* = 25).

The above analysis also revealed a significant interaction between action frame and inequality direction (*F*(1,24) = 14.605, p = 0.001). Follow-up permutation testing indicated that this was driven by more self-oriented behavior in the *Take* frame than in the *Give* frame (difference in mean inequality-direction effect  = 0.27, two-tailed p < 0.001). Since default offers of debt or pain to the other participant were higher in the *Take* frame, this effect is consistent with a *status quo* bias in dictator behavior. As described below a *status quo* bias was also evident in the mean offers across all three outcome modalities. The remaining interactions in the three-way ANOVA (between modality and action frame, and the three-way interaction) were not significant (*F*(1,24) = 0.660, p = 0.424 and *F*(1,24) = 0.604, p = 0.444 respectively).

### Status Quo Bia

Owing to the nature of the choices in this study, in which an initial allocation could either be accepted or altered, we expected to observe a *status quo* bias, reflecting an overall reluctance to change the current state of affairs^32^,^33^,^34^. A *status quo bias* predicts higher offers in the *Take* relative to the *Give* frame, since default offers are higher in the *Take* frame. As displayed in Fig. 6, mean offers in the *Take* frame were indeed significantly higher than those in the *Give* frame, for both pain (p < 0.001, two-tailed permutation test), money gains (p < 0.001) and money losses (p < 0.001).

### Modeling Dictator Behavior

In the above analyses, concepts such as inequality aversion and hyperaltruism are referred to without their being formally defined. To make the meaning of these concepts more explicit, and therefore less susceptible to logical error, we next compared alternative models of social utility in accounting for the observed choices. In particular we sought to test a simple form of social welfare preference that is capable of accounting for both hyperaltruism and inequality aversion. Under this model, termed social discounting, a person’s social utility is given by a weighted sum of their own utility and that of others, shown here for the two-person case:


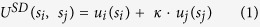


Here 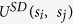 is a social utility function governing the utility to player *i* of an allocation with payoff *s*_*i*_ to player *i* and *s*_*j*_ to player *j*, *u*(*s*) is a utility function over individual payoffs and *κ* is a social discounting parameter which governs the contribution of player *j*’s payoff. If *u*(*s*) is concave for both gains and losses (decreasing marginal utility for gains and increasing marginal utility for losses/harms) then, for *κ* > 0 the above function predicts a tendency to spread the payoff across individuals. This has an intuitive interpretation as a motive to give to those most in need. Owing to concave utility, individuals with lower payoffs will value the same increase in their payoff more than individuals with higher payoffs.

Predictions of this model are displayed in Fig. 7, for the special case where *u*_*i*_ and *u*_*j*_ are concave and identical (the same form of utility function for payoffs to self and other). Where *κ* < 1, player *i* will assign more weight to their own payoff than those of the other player, and therefore is motivated to allocate more than half of the benefit to themselves. For *κ* = 1, players can maximize their utility by achieving equal payoffs (*s*_*i*_ = *s*_*j*_). For the opposite case of *κ* > 1, corresponding to hyperaltruism, player *i* is motivated to allocate more than half of the benefit to the other player.

We compared this model with an existing model based on social-welfare preferences, namely Charness-Rabin social preferences, which similarly assumes that people behave as if to maximize a weighted combination (in this case an average) of the payoffs of all players^20^. Rather than incorporating concave utility over individual payoffs, under Charness-Rabin preferences the weighted average is modulated by the existence of advantageous or disadvantageous inequality. We also tested two models based on social comparison, Fehr-Schmidt utility^6^ and the Equity, Reciprocity and Competition model (ERC)^5^. Under Fehr-Schmidt utility allocations are penalized in direct proportion to the degree of inequality (while allowing different slopes for advantageous and disadvantageous inequality) leading to linear inequality aversion. Under ERC squared deviations from even utility spreading are penalized, leading to increasing marginal inequality aversion.

Of the models tested, only the social discounting model can theoretically incorporate both hyperaltruism and increasing marginal inequality aversion, two key patterns observed in our data for pain. With the standard parameter bounds, Fehr-Schmidt utility accounts for neither finding, ERC accounts for non-linear inequality aversion, but not hyperaltruism and Charness-Rabin preferences are capable of incorporating hyperaltruism, but do not incorporate non-linear inequality aversion. We therefore hypothesized that the social discounting model would provide the best fit to the observed data. Since the behavioral data are consistent with a *status quo* bias, we modified each model to incorporate this effect, by assigning a cost to altering the *status quo*. Models were fitted on an individual subject basis by maximum likelihood, by means of a logistic function linking utilities with choice probabilities, and were compared using the Bayesian Information Criterion (BIC). The latter is an approximation to the Bayesian model evidence which favors models that account well for the observed data (in this case models with higher maximized likelihood), whilst penalizing model complexity (in this case models with more parameters). To reduce the number of possible models, money gains and losses were fitted jointly using the same set of parameters, which appeared justifiable given the relatively close correspondence of choices in these two conditions (Figs 5 and 6). (See Methods for detailed model specifications).

Consistent with predictions, the social discount model, with the addition of a *status quo* bias, was unequivocally the best fitting model at the group-level (highest maximum likelihood and lowest BIC) for both pain (Fig. 8a; BIC difference of 124 over the next best model, in this case Charness-Rabin social preferences with a *status quo* bias) and money (Fig. 9a; BIC difference of 152 over the next best model, in this case ERC with a *status quo* bias). The choice probabilities predicted by the model, formed by taking the mean of individual choice probabilities across subjects, are overlaid with the observed data for pain and money in Figs 8b and 9b respectively. For pain, mean *κ* = 1.17 (95% CI by resampling = 0.95–1.44; *N* = 47), indicating a non-significant trend at the group level towards assigning greater weight to others’ pain, i.e. hyperaltruism, in keeping with the behavioral findings above. For money, mean *κ* = 0.81 (95% CI by resampling = 0.64–0.96, one-sample p = 0.0162*; N* = 25), indicating significantly self-oriented preferences. Also in keeping with the preceding model-free analyses, mean *κ* for money was significantly lower than that for pain (p = 0.0158, two-tailed permutation test), supporting the conclusion of more altruistic choices for pain than for money.

### Evaluation of Experienced Pain Allocations

By generating equivalent outcomes framed as either *Non-Social* (no information given about the other player’s allocation) or *Social* (players were informed of the other’s allocation), where the latter can be framed as resulting either from the choices made by the dictator (*Social-Intentioned* condition) or from the play of chance (*Social-Chance* condition), we examined the effects of inequality and perceived intentionality on the perception of pain. We predicted that advantageous inequality would reduce and disadvantageous inequality increase pain perception. We also predicted that perceived intentionality, in the *Social-Intentioned* condition, would amplify this effect, resulting in an effect of intentioned advantageous inequality to reduce pain perception, termed a ‘kindness effect’ and an effect of intentioned disadvantageous inequality to increase it, termed a ‘meanness effect’.

Full ratings data was available from 50 subjects, 25 dictators and 25 receivers. We expected to find that receiving more shocks at a given current level would increase the rated intensity of the pain, owing to sensitization from one shock to the next. Indeed, a repeated measures ANOVA on intensity ratings across *Non-Social* with *Social-Chance* conditions across all subjects revealed a significant main effect of number-of-shocks (*F*(2,98) = 4.238, p = 0.017; partial *η*^2^ = 0.08). We predicted that priming inequality in the *Social-Chance* condition would increase the slope of this relationship, manifest as an interaction between condition and number of shocks. In fact we found no evidence for the predicted number-of-shocks by condition interaction (*F*(2, 98) = 0.045, p = 0.956), and no significant main effect of condition (*F*(1,49) = 0.297, p = 0.588), suggesting that inequality *per se* did not influence pain ratings (Fig. 10). However, comparison of intensity ratings from receivers (*N* = 25) in *Social-Chance* and *Social-Intentioned* conditions did show the predicted number-of-shocks by condition interaction (*F*(2, 48) = 11.657, p < 0.001, Greenhouse-Geisser corrected), as well as a main effect of number-of-shocks (*F*(2,48) = 8.026, p = 0.004, Greenhouse-Geisser corrected;) and no significant main effect of condition (*F*(1,24) = 1.218, p = 0.281), indicating that perceiving the other participant as responsible for shock allocations significantly influenced pain perception. Pairwise comparisons revealed this effect was driven by lower intensity ratings in the *Social-Intentioned* condition for 6 shocks (mean rating difference = 0.645 points on VAS, 95% CI of the difference [0.15 1.13]; *t*(24) = 2.72, p = 0.012, two-tailed paired test) whilst intensity ratings for 18 shocks did not differ significantly between the two conditions (mean rating difference = 0.046 points on VAS, 95% CI of the difference [−0.22 0.32]; *t*(24) = 0.351, p > 0.250), indicating a significant ‘kindness effect’, but no significant ‘meanness effect’ (Fig. 10).

Finally, despite the absence of a significant effect of inequality on pain perception at the group level, we nevertheless tested an hypothesis that a putative effect of inequality on pain perception in dictators might be a function of the degree of altruism exhibited in choices to allocate pain. In particular we predicted that less altruistic participants would show a greater effect of disadvantageous pain inequality on pain perception. However we found no significant correlation between dictators’ social discount factor, κ, and the difference in subjective pain ratings of 18 shocks between the *Non-Social* and *Social-Chance* conditions (Spearman rho = −0.11, p > 0.250).

## Discussion

Psychologists have long been interested in the circumstances under which people are willing to cause or relieve others’ pain. The experiments of Stanley Milgram (unacceptable by many ethical standards, including those of the time^35^) reported that under specific conditions, designed to encourage obedience to the wishes of the experimenter, people could be induced to cause high levels of pain to others^36^,^37^. However numerous studies that have hitherto attracted less attention illustrate that humans are generally reluctant to actively harm others^38^,^39^, show empathic responses to others’ pain^13^,^14^,^15^, and even choose to suffer pain, or pay money themselves to avoid causing pain to others^16^,^17^,^40^,^41^. These studies combine to suggest that people find others’ pain intrinsically aversive. Separately, economists have sought to quantify altruistic human behavior through formal games, finding that many people prefer to share *money* with others, even when there is no financial reward for doing so^10^, a behavior that has often been characterised as a preference for equality. Here we integrate these disparate lines of research, by formalizing the transfer of both pain and money between individuals in economic terms using a modified dictator game.

We found that being allocated more pain than others motivated people to transfer pain to others, while being allocated less pain than others motivated people to take on others’ pain, consistent with a preference for equal allocations of pain. Furthermore, increases in pain inequality had an increasing effect on the motivation to redress inequality. In psychological terms this can be viewed in terms of drive reduction: the further one is from a desirable goal, the stronger the motivation to act^42^,^43^. In economic terms this finding indicates an increasing marginal aversion to inequality^5^,^8^. We showed that this behavior can result from a more fundamental principle of diminishing marginal utility (or in psychological terms, satiety) if it is assumed that social utility is given by a sum of individual utilities, weighted by social discount factors. If people have some degree of concern for others, then diminishing marginal utility creates a motive to donate to those who have least, since they will benefit most from increases in utility^44^. For pain, the model assumes increasing marginal disutility, such that the motive to share pain arises directly from the escalating cost of pain to each individual. Put simply, those in greatest pain are seen as being in the greatest need of reductions in pain. An appealing feature of this model is that it derives a higher-order social preference (for equality) from a more basic psychophysical principle (satiety).

We termed the above model ‘social discounting’ since it is formally equivalent to conventional economic models of intertemporal choice, which combine an instantaneous utility function with a *temporal* discount factor^45^,^46^. Several previous reports have proposed that social and delay discounting can be considered formally equivalent, in support of which people discount delayed rewards and rewards given to individuals at increasing degrees of social distance from themselves according to the same hyperbolic form^47^,^48^,^49^,^50^,^51^. Furthermore, when asked to allocate reward, or relief from pain, over more than one time period, many people show a preference for spreading the benefits evenly^52^. This behavior can be accounted for by assuming diminishing marginal utility for rewards at each discrete time point, and as shown here is formally analogous to the tendency to spread utility across individuals^53^. An important direction for future research will be to examine whether these behaviors correlate across individuals, consistent with their hypothesized common origin in non-linear utility.

Unlike models based exclusively on social comparison, the social discounting model accounts for the observation that people will sacrifice decreases in equality for gains in efficiency (the total payoff to all players)^10^. However, in its current form the model cannot account for the opposite behavior, namely sacrifices in efficiency for the sake of achieving equality^54^. The model shown here might feasibly be extended to incorporate this behavior, by adding a direct social comparison term:


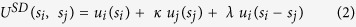


The first and second terms are exactly as shown in Equation 1. The first term corresponds to self-interest and the second to concern for others; together, through the concavity of *u*(*s*), these terms lead to a preference for spreading utility across individuals, which for egalitarian preferences (*κ* = 1, can be interpreted either as “my need is greater than yours”, in the case of disadvantageous inequality or “your need is greater than mine”, in the case of advantageous inequality. The third term embodies a specific benefit to having more than others (i.e. ‘competition’); by contrast to the first two terms, this term motivates actions which deprive others of benefit simply to improve one’s own relative standing, and corresponds to a specific meaning of ‘envy’ in psychodynamic theory^55^. A fourth term might also be added to represent the direct cost of being better off than others, however this is arguably unnecessary, since concrete instances of people deliberately destroying their own resources to make others feel better are relatively rare.

We found that, for monetary outcomes, people more frequently acted to correct disadvantageous as compared with advantageous inequality, as seen in previous findings where disadvantageous monetary inequality has a larger motivating effect^8^. For pain, however, we found no significant degree of asymmetry. Consistent with this participants were significantly more altruistic when in receipt of an allocation of pain than when allocating money. We also observed hyperaltruism for pain, whereby close to a third of participants (15 out of 47, 32%) retained more than half the shocks across all choices. This pattern was readily modeled by allowing the weighting given to others’ utility to exceed that given to one’s own utility. The prevalence of hyperaltruistic behavior seen here is closely comparable to that previously reported for a conventional dictator game with pain, which found that 16 out of 54 (30%) participants allocated less than half of the painful cold immersion time to the other participant^18^. (Notably both proportions are markedly lower than the prevalence of hyperaltruism in a recent study which required participants to trade off pain and money, where over half of participants displayed hyperaltruism^19^. One explanation for this discrepancy would be that, in addition to a baseline hyperaltruistic tendency for pain in some individuals, people are specifically reluctant to accept monetary profit from others’ pain).

Why people appear to show greater altruism when allocating pain as compared with money remains unclear. Our findings demonstrate a reduced form estimate of this effect. We do not identify structural estimates of the impact of various parameters, but we can speculate about mechanisms based on these data. One possibility is that, while people frequently exchange money in everyday life, exchanging pain is more unusual. This might lead people to be be more uncertain about others’ responses to pain compared to their responses to money and therefore behave more altruistically when allocating pain as a precaution against provoking an unexpectedly severe response^19^. Of course the current experiment does not permit reciprocity, but it seems likely that altruism in dictator games in part reflects the accumulated experience of participants that social interactions are almost always reciprocal^56^. A second possibility is that a particular aversion to causing pain in others might arise in a social norm effect. Though there are many circumstances under which it is acceptable to deprive others of money, causing physical harm to others is rarely deemed acceptable. Finally it is possible that the findings here do not truly reflect intrinsically greater altruism for pain than for money, but are peculiar to our experimental scenario. For example, participants might have wished to signal to the experimenter or to the other participant that they were capable of enduring pain, or that they were particularly charitable. In monetary dictator games obscuring the link between the dictator’s choices and the resulting outcomes engenders more selfish behavior^12^,^56^,^57^,^58^, and further work ought to examine whether the apparent increase in altruism seen for pain survives in cases where the dictator’s actions are less transparent, for example where random variability is added to the dictator’s offers^59^. Again, these are at present speculations and we hope they stimulate further research to unpick the underlying mechanisms. We note that a previous study with a different design also finds greater altruism for pain than money^18^, though similar issues might apply to this finding also.

Finally, by implementing selected allocations for real, we were able to examine the effects of inequality and perceived intentionality on the perception of pain itself. Although we found no significant influence of inequality *per se* on pain perception, we did observe a ‘kindness effect’, whereby participants rated a low number of shocks as less intense when they believed that the other participant had chosen to accept a high number of shocks at the same time, thus enduring extra pain for their benefit. This finding may reflect participants’ perception of being in the presence of a supportive other, consistent with existing findings that the presence of a familiar conspecific reduces pain responses in both humans^60^ and animals^61^,^62^ Lending support to this idea, one previous study found that pain chosen by another participant was rated as less severe if the receiver believed that the other participant would increase their (the receiver’s) monetary payment by giving pain, rather than believing that the other had no apparent motive for giving pain^30^.

Unlike previous studies we found no evidence for the converse (‘meanness’) effect, namely an effect of selfish behavior on the part of the dictator to increase pain perception for the receiver^30^,^31^. An explanation for this null result might be that in the current study intentioned-inequality was in the number of shocks; effects of intentioned-inequality on subjective intensity ratings might therefore be expected to resemble the effect of increasing or decreasing the *number* of shocks. Subjective intensity ratings did increase as a function of the number of shocks (consistent with sensitization), however this relationship saturated at higher numbers of shocks. Under this account a meanness effect would be equivalent to increasing the number of shocks from an already high level, with little resulting effect on pain perception. To formalize this hypothesis we modeled intensity ratings as a concave psychophysical function of number of shocks, using a Weibull function with two parameters, asymptote, *A*, and latency, *L*, constrained to pass through the origin:


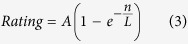


Least squares fits of this function, displayed in Fig. 10, illustrate that the saturation hypothesis appears plausible.

A key question for future research is whether relative deprivation or suffering in one modality alters responses to inequality in other modalities. There is already evidence to support this suggestion. For example, Mancini and colleagues report that experiencing pain increases self-oriented behavior in a one-shot ultimatum game^54^, and that, somewhat in opposition to the kindess effect shown here, perceived monetary unfairness reduces neural responses to pain^63^. Such generalization across domains is not incongruent with a wider societal observation that relative poverty correlates with both violent and acquisitive criminal behavior^3^,^64^,^65^.

## Materials and Methods

### Participants and Role Allocation

We recruited 78 participants (46 female) from the University College London Psychology Subject Pool. A power calculation based on a Student’s *t*-distribution indicated that a sample size of 34 would be required to detect a medium sized effect (Cohen’s *d* = 0.5), while a sample size of 15 would be required to detect a large effect (Cohen’s *d* = 0.8) at 80% power^66^. The primary purpose of this study was to examine how people choose to distribute pain, and their responses to unequal allocations of pain. We sought to examine whether hyperaltruism and inequality aversion would both manifest in such choices. We therefore aimed to elicit pain dictator game choices from at least 35 participants. The final sample size easily exceeded this total (*N* = 47; 29 female, mean age 27.7 years, s.d. 8.5 years).

Given that the experimental design differs from a standard dictator game setup, we tested the same design with monetary choices, to allow fair comparison between responses to pain and money inequality. We therefore elicited monetary choices from a subset of the same participants who were willing and available to take part in this component of the study. A monetary questionnaire was issued at a later date to these 47 participants; here the sample size was determined by the number responding to this request (*N* = 25; 16 female, mean age 29.6 years, s.d. 11.6 years). Of the 47 participants completing pain dictator game games, 31 took part in the experiential component of the study. For this component, we separately recruited 31 participants to act as ‘receivers’. Due to experimenter error, datasets from 6 of these 31 pairs were incomplete, resulting in full experiential data from 25 pairs (*N* = 50; 30 female, mean age 27.8, s.d. 10.2). Dictators and receivers did not actually meet each other, but each was briefed that they would be interacting with another participant in an adjacent testing room through an intranet link; they were informed that there was no opportunity for reciprocity. Experiments were carried out at the Wellcome Trust Centre for Neuroimaging, University College London. Participants were compensated for their participation at a rate of £10 per hour.

### Ethics Statement

All participants gave full informed consent prior to the experiment. After the experiment participants were debriefed and given the opportunity to provide feedback. The study procedure was approved by the UCL Research Ethics Committee (Ethics no. 3953_001), and procedures were carried out in accordance with the approved guidelines.

### Pain Stimuli

Throughout the experiment each participant sat in front of a computer monitor while choice options were presented on-screen, and indicated their decisions using the keyboard. The presentation package COGENT 2000 (University College London) was used for stimulus presentation and response acquisition. Electrical stimuli were delivered using a DS5 Digitimer (Letchworth Garden City, England) constant current stimulator. A single “shock” consisted of a train of seven 10ms square-wave pulses delivered at 40ms intervals. When multiple shocks were delivered these were spaced 250 ms apart. After providing consent, participants underwent a standardized thresholding procedure, which allowed selection of shock intensities corresponding to an equivalent subjective level of discomfort for each participant^67^,^68^. Participants were first asked to rate the pain associated with shocks of varying intensities using a 10-point visual analogue scale (VAS). Firstly two iterations of a stochastic increasing current staircase procedure were used to identify the maximum intensity the participant felt they could tolerate for the purposes of the experiment. Participants were reassured that during the experiment all stimuli would be below their maximum tolerance. Secondly participants rated a randomized sequence of sub-maximal shock intensities. The least-squares fit of a psychophysical function (a three-parameter Weibull distribution) describing the subjective ratings at each current level was determined for each participant, and inverted to determine current levels (in mA) for use during the experiment, which corresponded to ratings of 5/10 and 8/10 respectively.

### Dictator Game Choices

**Figure 1:**
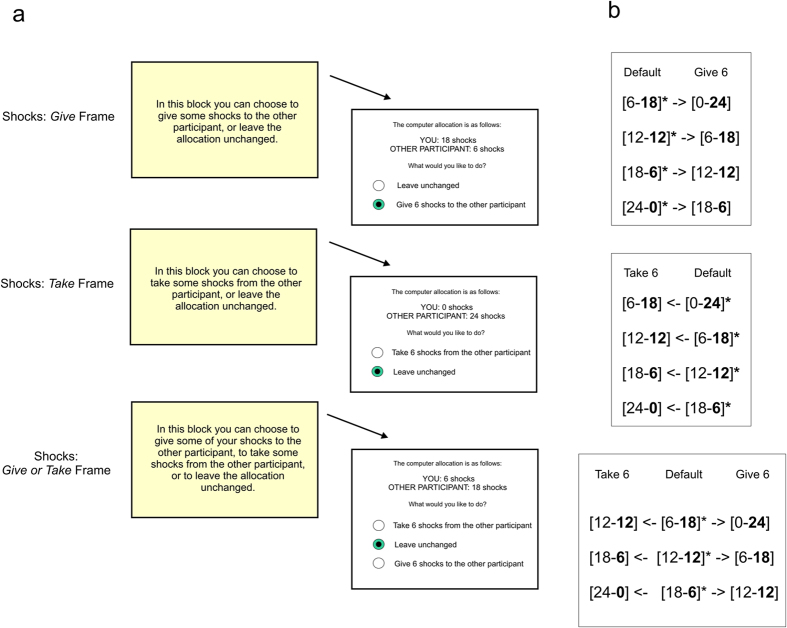
Modified Pain Dictator Game. (**a**) In each of three possible action frames the dictator was given the opportunity to alter or leave unchanged default allocations of 24 brief, moderately painful, cutaneous electric shocks between themselves and another participant, termed the Receiver. In a *Give* frame the dictator could choose to either give six of their allocated shocks to the Receiver, or leave the allocation unchanged. In a *Take* frame the Dictator could choose to either take on six of the Receiver’s shocks, or leave the allocation unchanged. In a *Give or Take* frame both giving and taking options were available, as well as the option to leave the default unchanged. (**b**) Choice options within each frame. Allocations are in the format [shocks for Dictator—shocks for Receiver]. The number of shocks allocated to the Receiver, highlighted in bold, was termed the ‘offer’. Asterisks indicate the default allocation (which would result if participants chose ‘leave unchanged’). We also implemented equivalent choices for money, in which shocks were simply replaced with sums of money in pounds sterling (money gain condition) or with amounts of debt in pounds sterling (money loss condition). Since debt in the money loss condition was subtracted from an endowment of £24, the net allocations under the two conditions were equivalent. In both forms of the task a selection of choices were implemented.

Dictator game choices were elicited through an online interface (Qualtrics.com; Provo, UT); on each the computer suggested a default allocation of 24 shocks between dictator and receiver, and the dictator chose whether to alter this allocation, or leave it unchanged (Fig. 1). Dictators were informed that the subjective intensity level of the shocks would vary between 4/10 and 8/10 on a VAS, but that the subjective level for themselves and the other player would always be the same on a given trial. In a *Give or Take* condition default allocations (number of shocks for the dictator listed first) were [18–6], [12–12] or [6–18]. The dictator chose whether to give 6 shocks to the receiver from their own allocation, to take 6 shocks from the receiver’s allocation (which would be added to their own allocation) or to leave the allocation unchanged. In a *Give* condition default allocations were [24–0], [18–6], [12–12] or [6–18]. The dictator chose whether to give 6 shocks of their shocks to the receiver or to leave the default allocation unchanged. In a *Take* condition default allocations were [18–6], [12–12], [6–18] or [0–24]. The dictator chose whether to take 6 shocks from the receiver’s allocation, or to leave the allocation unchanged. Notably equivalent outcomes were attainable in all frames. Each choice option was presented twice, creating 22 choices in total. Dictators were informed that a selection of their choices would be played for real in the experiential part of the experiment, should they consent to take part. The order of conditions and trials within conditions was randomized.

In a follow-up experiment we elicited equivalent choices with money in a subset of the same participants, where the numbers of shocks were replaced with quantities of money in pounds sterling. In a Money Gain condition participants were told that they had won a prize of £24 between themselves and the other participant. On each choice the computer suggested a default allocation for the money and the dictator chose to either alter this allocation, or leave it unchanged. In a Money Loss condition participants were informed that they and the other participant had *each* won a prize of £24, however that they must also pay a debt of £24 between them both. Net allocations in the Money Loss condition were equivalent to the Money Gain condition. Participants were informed that we would randomly select some choices from the entire set of choices made by all participants to be paid out for real, which we honored.

### Experiential Phase

**Figure 2:**
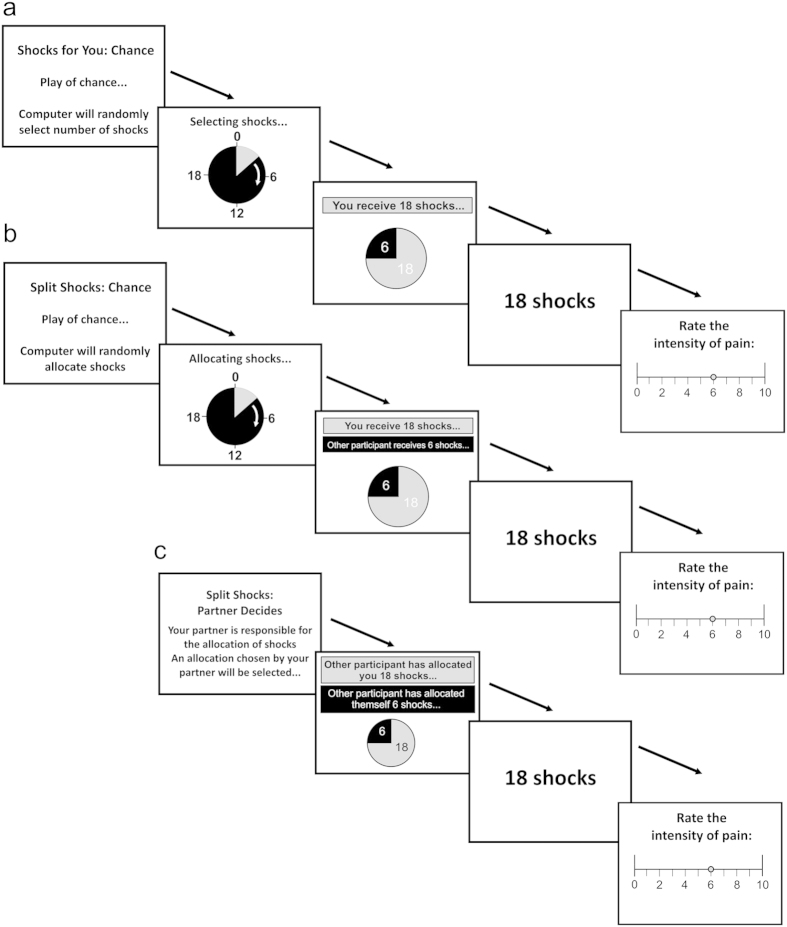
Experiential Phase: Trial Structure for Receivers. (**a)** On *Shocks for you: Chance* trials participants were informed that the outcomes would only be relevant to them, and did not involve the other participant. The computer then selected an allocation of shocks at random. The participant received the stated number of shocks and was then asked to rate the intensity of the shocks on a visual analogue scale. We subsequently elicited participants’ willingness to pay to avoid three further repeats of the same outcome (not shown in this figure). (**b**) *Split Shocks: Chance* trials proceeded in the same manner, though in this case the computer ‘randomly’ chose how to allocate 24 shocks between the two participants. (**c**) The trial structure was identical in *Split Shocks: Partner Decides* trials, though on these trials the Receiver was informed that an allocation chosen by their partner would be selected and played for real.

**Figure 3:**
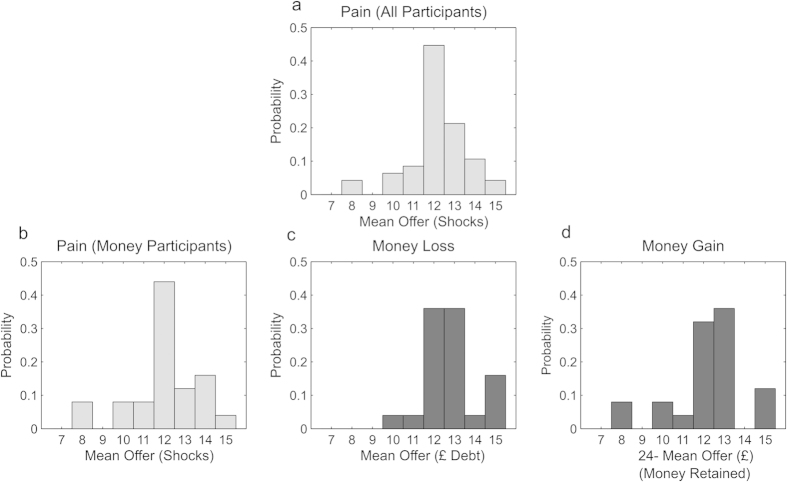
Distribution of Mean Resulting Dictator Game Offers. Histograms summarizing the distribution of mean resulting dictator game offers, averaged across all choices made by each participant. In each case the horizontal axis represents an increasing measure of self-oriented behavior. (**a**) Pain dictator game, mean offers of shocks by all 47 participants, (**b**) pain dictator game, mean offers of shocks made by 25 participants who also completed monetary choices, (**c**) money dictator game, mean offers of debt in the loss frame, (**d**), money dictator game, mean money retained by the dictator in the gain frame. Offers are out of a total endowment of 24 shocks (**a**,**b**) or £24 (**c**,**d**).

**Figure 4:**
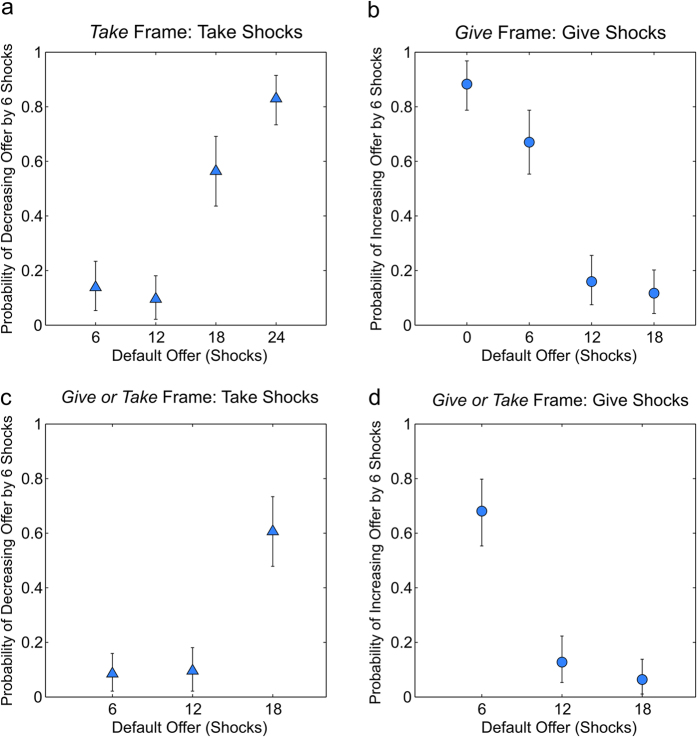
Dictator Behavior for Pain as a Function of Default Allocations. For each plot a single data point represents a mean choice probability across 47 participants; error bars are 95% confidence intervals around the mean, generated by re-sampling the data 100,000 times with replacement. (**a**) The probability, in the *Take* frame, that dictators chose to take 6 shocks from the receiver’s allocation (decreasing the resulting offer by 6 shocks), as opposed to leaving the allocation unchanged, is plotted as a function of the initial allocation of shocks to the receiver (the default offer). Dictators were increasingly likely to take shocks from the receiver as the initial allocation to the receiver increased, consistent with an increasing marginal cost to having fewer shocks than the receiver. (**b**) The probability, in the *Give* frame, that dictators chose to give 6 of their allocated shocks to the receiver (increasing the resulting offer by 6 shocks) is plotted as a function of the initial allocation of shocks to the receiver (the default offer). Dictators were increasingly likely to give shocks to the receiver as the initial allocation to the receiver decreased, consistent with there being an increasing marginal cost to having more shocks than the receiver. Similarly, in the *Give or Take* frame, the probability that dictators chose to take 6 shocks from the receiver’s allocation increased as the initial allocation to the receiver increased (**c**), and the probability that dictators chose to give 6 of their shocks to the receiver decreased as the initial allocation to the receiver increased (**d**).

**Figure 5:**
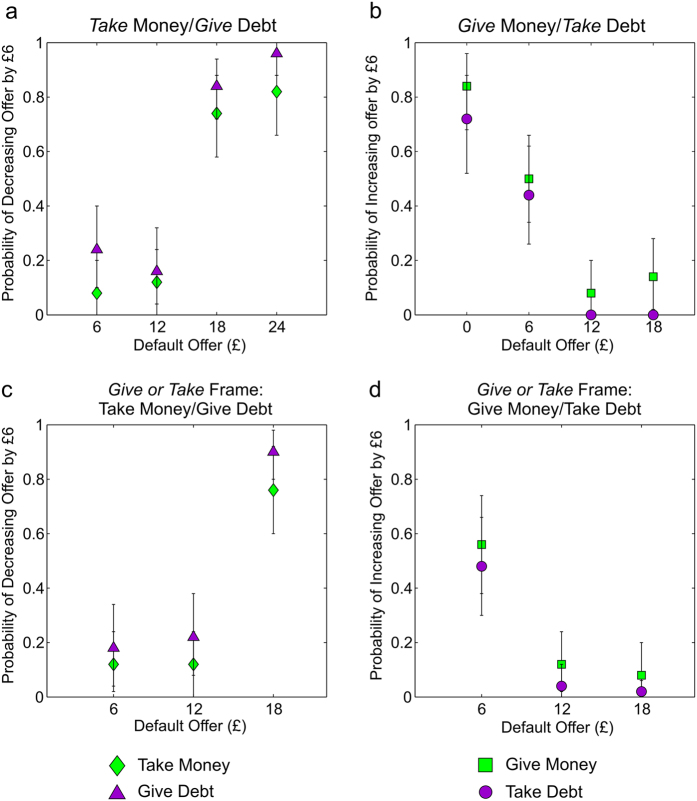
Dictator Behavior for Money as a Function of Default Allocations. For each plot a single data point represents a mean choice probability across 25 participants; error bars are 95% confidence intervals around the mean, generated by re-sampling the data 100,000 times with replacement. (**a**) The probability (in the *Take* frame) that dictators chose to *take* £6 from the receiver, as opposed to leaving the offer unchanged, is plotted as a function of the initial net (default) offer, indicated by green diamonds. Also plotted is the probability (in the *Give* frame) that dictators chose to *give* £6 of debt to the receiver, indicated by the purple triangles. In both cases, dictators were increasingly likely to reduce the offer as the initial offer increased, consistent with there being an increasing marginal cost to having less money than the receiver. (**b**) The probability (in the *Give* frame) that dictators chose to give £6 to the receiver, as opposed to leaving the offer unchanged, is plotted as a function of the net default offer, indicated by green squares. Also plotted is the probability (in the *Take* frame) that dictators chose to take £6 of debt from the receiver, indicated by purple circles. In both cases, dictators were increasingly likely to increase the offer as the initial offer decreased, consistent with an increasing marginal cost to having more money than the receiver. Similarly, in the *Give or Take* frame, the probability that dictators chose to decrease the net offer by £6, either by taking money (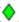) or giving debt (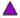) increased as the initial offer increased (**c**), and the probability that dictators chose to increase the net offer by £6, either by giving money (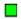) or taking debt (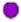) decreased as the initial offer increased (**d**). A tendency towards self-oriented choices is evident from the greater propensity to decrease rather than increase offers, for the same degree of inequality.

**Figure 6:**
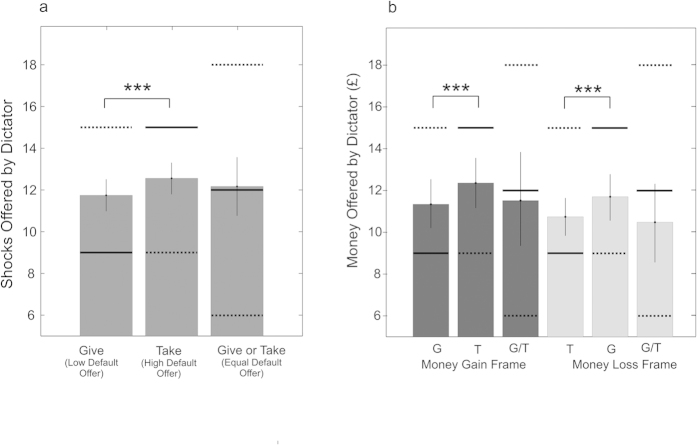
A Status Quo Bias in Dictator Behavior. (**a**) Group-level mean offers of shocks across all dictators (vertical axis; *N* = 47), for each action frame (horizontal axis category). Solid black horizontal bars indicate the mean default offer under each frame. Upper dashed horizontal bars show the mean offers that would result from maximally selfish behaviour, lower dashed horizontal bars the mean offers that would result from maximally selfless behaviour. Mean offers were significantly higher in the *Take* frame than in the *Give* frame, consistent with a *status quo* bias. (**b**) Group-level mean net offers of money across all dictators (vertical axis; *N* = 25) under *Give* (G), *Take* (T) and *Give or Take* (G/T) action frames (horizontal axis category, individual bars), under both Gain and Loss (debt) frames (horizontal axis groupings). Here, taking money is equivalent to giving debt, and vice versa. Solid black horizontal bars indicate the mean default offer under each frame. Upper dashed horizontal bars show the mean offers that would result from maximally selfless behaviour, lower dashed horizontal bars the mean offers that would result from maximally selfish behaviour. A *status quo* effect is evident for money gains and losses (debt), whereby net offers were higher when the net default offer was high (*Take* frame for money gains and *Give* frame for money losses), compared with when the net default offer was low (*Give* frame for money gains and *Take* frame for money losses). Error bars indicate 95% confidence intervals generated by resampling the data 100,000 times with replacement. Asterisks indicate p values associated with two-tailed permutation tests; ***p<0.001.

**Figure 7:**
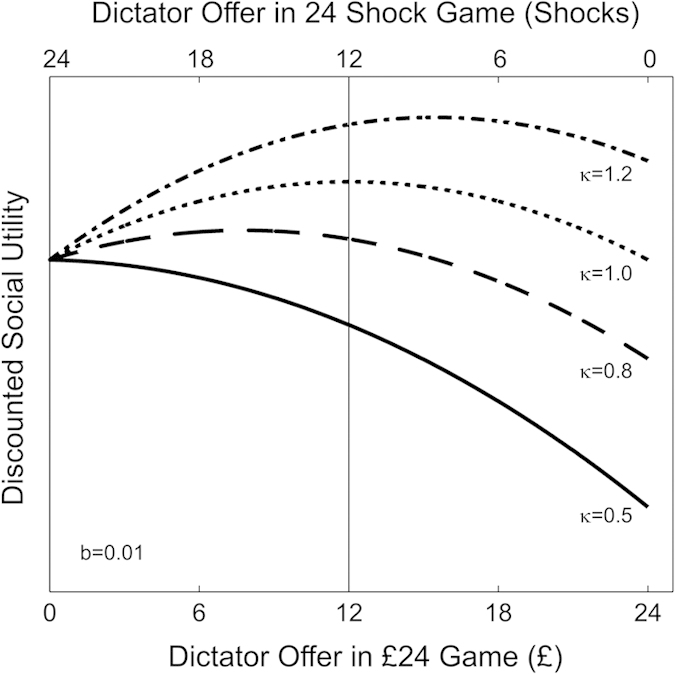
Social Utility Functions Based on a Social Discounting Model. The total utility predicted by a social discounting model of the form shown in Eq. 1 (vertical axis, arbitrary units), is plotted as a function of the offer to the receiver (*s*_*j*_) in a dictator game with an endowment of 24 units (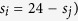. Two possible scenarios are shown, either a decreasing offer of painful shocks (horizontal axis, upper scale) or an increasing offer of money (horizontal axis, lower scale). The four lines represent different settings of the social discount parameter, *κ*, as labeled. In each case, 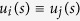, both given by the quadratic function 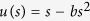, where 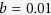. For *κ* = 1 the function has a maximum when allocations to both participants are equal 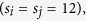 corresponding to symmetric inequality aversion. *κ* < 1 generates a preference for assigning more than half the benefit to oneself, while *κ* > 1 (hyperaltruism) generates a preference for assigning more than half the benefit to the receiver. For each parameter setting, moving away from one’s preferred allocation carries a non-linear (increasing marginal) cost.

**Figure 8:**
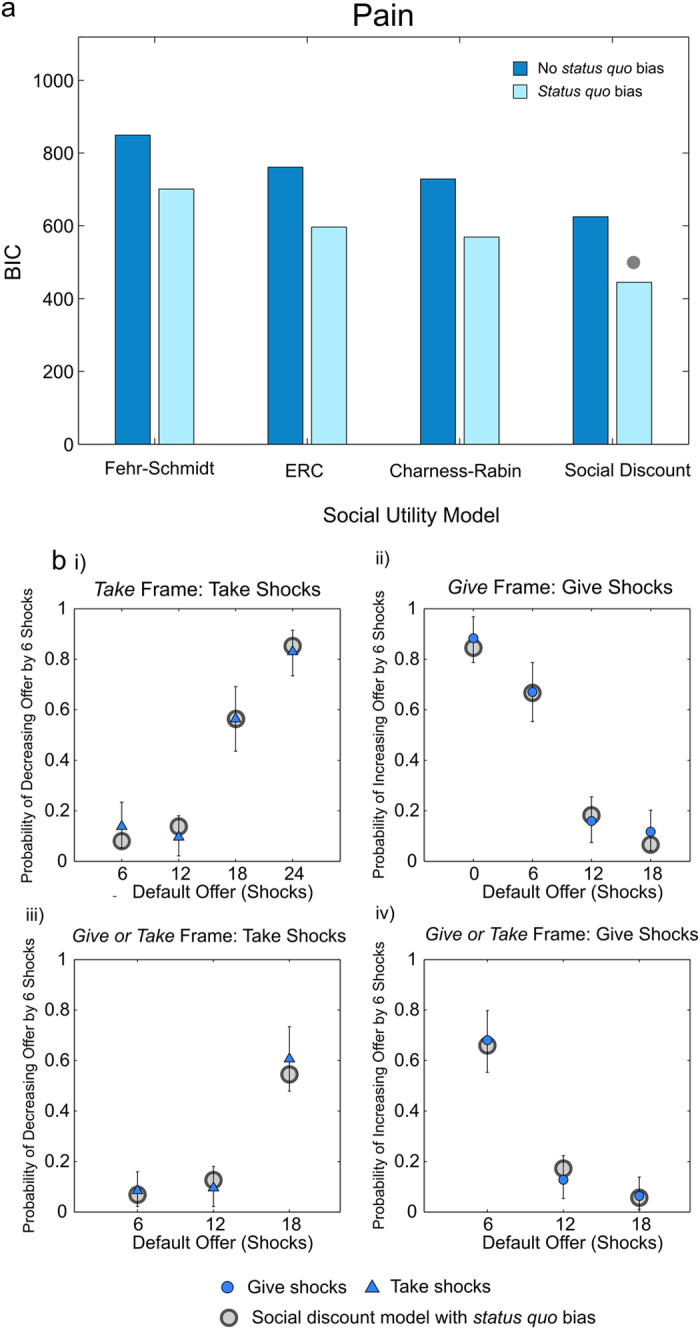
Model Fits to Pain Dictator Game Choices. (**a**) BIC values for the alternative models, either with *status quo* bias (light blue bars, right bar of each pair) or without (dark blue bars, left bar of each pair). The social discounting model provided the most parsimonious fit, indicated by the grey dot. (**b**) Maximum likelihood choice probabilities of the social discounting model, formed by taking the mean of the predicted choice probabilities across participants, are indicated by gray circles, and are overlaid with the observed choice probabilities as displayed in Fig. 4, indicated by blue triangles for choices to take on pain and blue circles for choices to give pain. As in Fig. 4, a single data point represents a mean choice probability across 47 participants and error bars are 95% confidence intervals around the mean, generated by re-sampling the data 100,000 times with replacement.

**Figure 9:**
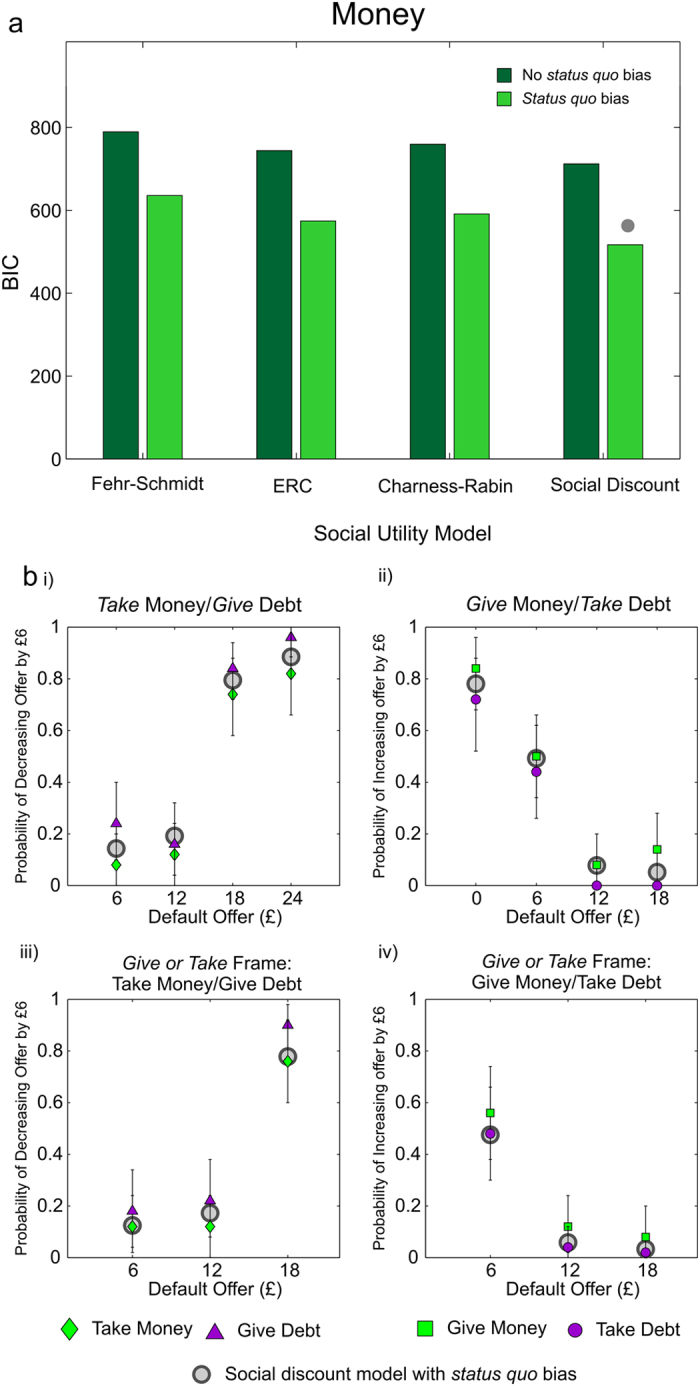
Model Fits to Money Dictator Game Choices. (**a**) BIC values for the alternative models, either with *status quo* bias (light green bars, right bar of each pair) or without (dark green bars, left bar of each pair). The social discounting model provided the most parsimonious fit, indicated by the grey dot. (**b**) Maximum likelihood choice probabilities of the social discounting model, formed by taking the mean of the predicted choice probabilities across participants, are indicated by gray circles, and are overlaid with the observed choice probabilities as displayed in Fig. 5, indicated by green diamonds for choices to take money, purple triangles for choices to give debt, green squares for choices to give money and purple circles for choices to take on debt. As in Fig. 5, a single data point represents a mean choice probability across 25 participants and error bars are 95% confidence intervals around the mean, generated by re-sampling the data 100,000 times with replacement.

**Figure 10:**
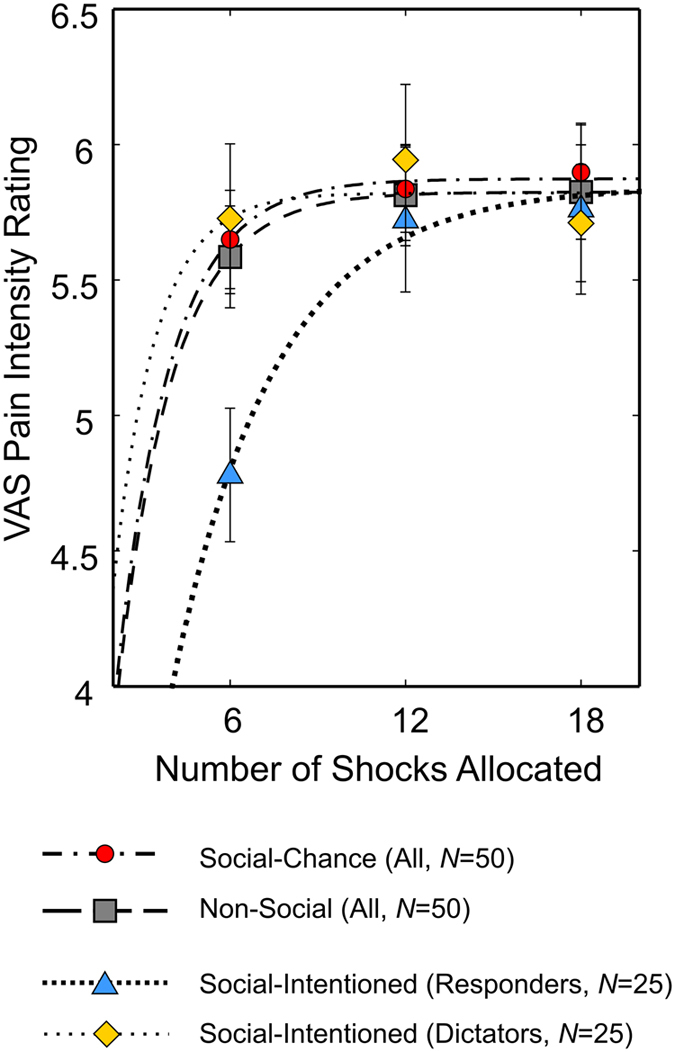
Rated Pain Intensity. Mean pain intensity ratings across participants on a 10-point visual analogue scale (VAS) for the Non-Social condition (all participants, N = 50, gray squares) the Social-Chance condition (all participants, N = 50, red circles) and the Social-Intentioned condition (split into dictators, indicated by yellow diamonds and receivers, indicated by blue triangles, N = 25 in each group). Data are modelled as a concave psychophysical function of number of shocks (dashed and dotted lines, see Supporting Results). Error bars represent one standard error above and below the mean. In the Social-Intentioned condition, when dictators behaved altruistically, allocating only 6 out a possible 24 shocks to the receiver, this significantly reduced pain perception relative to the other two conditions.

To examine the effects of inequality and intentionality on the subjective experience of pain, selected shock allocations were played for real in the laboratory. This phase consisted of three types of trial (Fig. 2). On ‘Shocks for You: Chance’ (the *Non-Social-Chance* condition) trials participants were informed that they would receive either 6, 12 or 18 shocks, which would be randomly assigned to them by the computer. On ‘Split Shocks: Chance’ trials (the *Social-Chance* condition) participants were informed that 24 shocks would be split between themselves and the other participant, according to one of three possible allocations [6–18], [12–12] or [18–6], which would be randomly selected by the computer. Finally a third type of trial (forming the *Social-Intentioned* condition) was referred to as ‘Split Shocks: You Decide’ for the dictator and ‘Split Shocks: Other Player Decides’ for the receiver. The receiver was informed that the dictator had chosen allocations of shocks in advance, and that on each trial of this type one of the dictator’s chosen allocations would be implemented. Dictators were informed that on each trial of this type one of their previously chosen allocations would be implemented, and that each allocation may be implemented more than once.

Both players were told that the intensity of the shocks could vary from trial to trial and that whilst they would not be told the intensity, the subjective level of the shocks for themselves and their partner would always be the same (both players would be told the *number* of shocks they were due to receive). All dictators made several choices resulting in the three allocations 6–18, 12–12 and 18–6 shocks. In fact, so as to control for context effects in the perception of pain, these three outcomes were selected with equal probability in both the *Social-Intentioned* and *Social-Chance* conditions. Similarly in the *Non-Social* condition, participants received 6, 12 or 18 shock with equal probability. Although participants were not informed of this sampling process, they were otherwise given faithful information regarding the setup.

The three trial types (conditions) were interleaved in 6 short blocks of 12 trials each. Within each condition, the three possible allocations of shocks were repeated twice at each of two different intensity levels (5/10 and 8/10). The order of conditions and of trials within each condition was randomly counterbalanced. The outcome allocation on each trial was indicated by a pie chart. In *Non-Social* and *Social-Chance* conditions, this was preceded by a “roulette-wheel” effect, emphasizing that the computer was randomly selecting the shocks. After the outcome was displayed shocks were administered to both players accordingly.

In the first session, after shock delivery each player rated the intensity of the shocks on a visual analogue scale. Immediately afterwards participants’ willingness-to-pay to avoid the shocks was obtained by means of a first-price “auction” (not shown in Fig. 2, see Supporting Results)^69^. Participants were endowed with 40 pence on each trial and were asked to indicate their willingness to pay from this endowment to avoid three repeats of their allocated shocks. On one in ten randomly selected trials their bid was compared to a market price, sampled from a uniform distribution in the range of 0–40 pence. If the participant’s bid exceeded the market price, they would avoid repeated instances of the allocated shocks, and would pay the amount they bid; otherwise they would receive three repeats of their allocation of shocks and would pay nothing. Participants took home as a “bonus” any money which they did not spend on these auction trials. Participants were informed that their bids only affected their own shocks, and did not alter the other participant’s shocks. In a second session, participants rated the fairness of the outcomes, the extent to which they perceived the other player to be responsible for the allocation and the extent to which they felt inclined to punish the other player for the allocation, each on a VAS ranging from 0 to 100 (see Supporting Results).

### Statistical Tests and Significance Thresholds

To test statistical hypotheses without making assumptions about the sampling distribution of the data we generated sampling distributions non-parametrically, by permutation testing. For one-sample tests this entailed randomly resampling the data with replacement, for two sample tests this entailed randomly reassigning the observed data to either of the comparison condition and computing the sample mean difference. To construct the sampling distribution 100,000 samples were taken in each case. We performed two-tailed tests throughout, with a standard significance threshold of α = 0.05. Where relevant we corrected for multiple comparisons by Bonferroni correction.

### Model Specification and Fitting

We tested alternative social valuation models for the choices made by the dictators. Specifically, we tested the social discounting model described above against three existing social utility models: Fehr-Schmidt utility^6^, Charness-Rabin social preferences^20^, and the Equity, Reciprocity and Competition model (ERC)^5^. The social discounting model assumed the form shown in Eq. (1). The utility functions over individual payoffs were assumed to be identical, such that 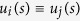, both given by the quadratic function 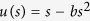, where *b* = 0.01.

The Fehr-Schmidt model penalizes unequal allocations, with separate penalties for advantageous inequality and disadvantageous inequality:


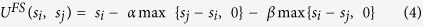



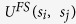
 is a social utility function governing the utility to player *i* of an allocation with payoff *s*_*i*_ to player *i* and *s*_*j*_ to player *j*, and *α* parameterizes the linear effect of disadvantageous inequity, *β* the effect of advantageous inequity. As is conventional, parameter bounds were set such that *α* ≥ 0 and 0 ≤ *β* ≤ 1.

The ERC model also implements a penalty for unequal payoffs, though in a non-linear manner, achieved by penalizing squared deviations from an even distribution of payoffs:


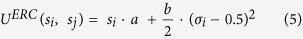


where *σ*_*i*_ is player *i*’s share of the total payoff:


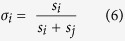


The parameter *a* weights self-oriented preferences, while *b* determines the contribution of inequality aversion; both parameters were bounded to be zero or positive.

Charness-Rabin social preferences assume that people behave as if to maximize a flexible weighted average of the payoffs of all players, where the weightings reflect the existence of advantageous or disadvantageous inequity as well as the effect of intentionality. For the two-person case:


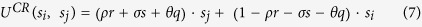


where


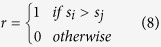



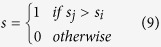



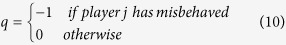


In this case *q* was set to zero, making *θ* redundant; the remaining weighting factors *ρ* and *σ* were bounded between zero and one.

All four models above were modified by adding a *status quo* bias, simply a fixed cost, *x*, associated with changing the allocation, such that for each model:


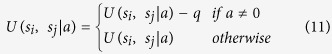


where *a* = 0 denotes accepting the default.

Model fitting was by maximum likelihood estimation; a standard logistic sigmoid (softmax function) was used to transform the social utilities of the allocations resulting from different actions into corresponding probability of choosing a particular action *a*, over alternative actions *a*′:


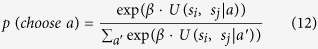


Here *β* is the inverse temperature parameter, equivalent to a logistic regression weight. With higher values of *β* behavior becomes more deterministic for choosing the allocation with higher utility. Constrained non-linear optimization was used in Matlab (Mathworks, MA, USA), to find model parameters which minimized the negative log likelihood of subjects’ choices. To avoid convergence on local minima, the optimizer was run within a random multi-started overlay with 1000 starting points drawn from a uniform distribution within the parameter bounds (RMsearch). Models were compared using the Bayesian Information Criterion (BIC), which is an approximation to the Bayesian model evidence, favoring models that provide a close correspondence to the data while penalizing model complexity^70^:


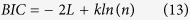


where *L* is the maximized group level log likelihood (formed by summing individual log likelihoods), *k* is the total number of fitted model parameters (summed across subjects) and *n* is the total number of fitted data points (summed across subjects). Lower values of BIC indicate a more parsimonious model fit.

### Survey Measures

Participants also completed a 20-item Paranoia Scale^71^, a 40-item Empathy Quotient questionnaire^72^, a Justice Sensitivity Scale (Victim and Perpetrator sensitivity)^73^ and a Vengeance Scale^74^ (see Supporting Results).

## Additional Information

**How to cite this article**: Story, G. W. *et al.* Social redistribution of pain and money. *Sci. Rep.* **5**, 15389; doi: 10.1038/srep15389 (2015).

## Supplementary Material

Supplementary Information
