# Supplementary material for: Social redistribution of pain and money
Source: Sci Rep. 2015 Oct 30;5:15389. doi: 10.1038/srep15389 (PMC4626774; doi:10.1038/srep15389)
Supplement: Supplementary Information [file srep15389-s1.doc]

Social redistribution of pain and money

Giles W. Story^a,b^, Ivo Vlaev^c^, Robert D. Metcalfe^d^, Molly J. Crockett^b,f^, Zeb Kurth-Nelson^e^, Ara Darzi^a^, Raymond J. Dolan^b^

^a^Centre for Health Policy, Institute of Global Health Innovation, Imperial College London, London, 10^th^ Floor, St. Mary’s Hospital, London, W2 1NY, UK

^b^Wellcome Trust Centre for Neuroimaging, University College London, London, WC1N 3BG UK

^c^Warwick Business School, The University of Warwick, Coventry, CV4 7AL, UK

^d^Becker Friedman Institute, University of Chicago, 575 S. University Ave., Chicago, IL 60637, US

^e^Max Planck UCL Centre for
Computational Psychiatry and Ageing Research, UCL, c/o Wellcome Trust Centre for Neuroimaging, University College London, London, WC1N 3BG UK

^f^Department of Experimental Psychology, University of Oxford, 9 South Parks Road, Oxford OX1 3UD, UK

Corresponding Author:

Dr Giles Story, Centre for Health Policy, St Mary's Hospital, London W2 1NY, UK

E-mail: [g.story@imperial.ac.uk](mailto:g.story@imperial.ac.uk), [g.story@ucl.ac.uk](mailto:g.story@ucl.ac.uk)

Tel : +44 (0)20 3448 4362

**Supporting Results**

**Willingness to Pay to Avoid Repeated Shocks**

In the first session, after shock delivery each player rated the intensity of the shocks on a visual analogue scale. Immediately afterwards participants’ willingness-to-pay to avoid the shocks was obtained by means of a first-price “auction”. We predicted a similar set of effects as observed for the responders in their willingness-to-pay (WTP) to avoid three further instances of the painful outcome on each trial. This was also expected to be the case as participants indicated their WTP immediately *after* rating the intensity of the shocks, allowing their ‘bids’ to anchored to their intensity ratings. However, whilst we observed a significant main effect of number-of-shocks on responders’ (*N*=25) WTP (*F*(2,48) = 15.89, p<0.001, Greenhouse-Geisser corrected) we observed neither a significant main effect of condition (*F*(2,48) = 0.202, p=0.756) nor a condition by number-of-shocks interaction (*F*(4,96) = 0.823, p = 0.513), suggesting that the motivational value of shocks was not influenced by the social context.

**Ratings of Fairness, Responsibility and Inclination to Punish.**

In a separate experimental block, participants also made ratings in percentage terms of how much they perceived the other participant as responsible for the allocation, how fair they perceived the allocation to be and the extent to which they felt inclined to punish the other participant. The results are shown in Figure S1. For responsibility ratings made by responders (*N*=25) there was a large significant main effect of condition (*F*(2,48) = 157.6, p<0.001; partial
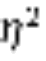
= 0.868), indicating a high degree of credibility in the experimental manipulation (Figure S1a). As expected participants rated receiving 18 shocks as less fair than receiving either 12 or 6 shocks, whilst the latter two outcomes were rated as equally fair when collapsing across conditions (Figure S1b; estimated marginal mean rating for 18 shocks = 30.8%, 95% CI [21.5-40.2], 12 shocks = 63.9% [54.2-73.6], 6 shocks = 61.3% [52.5 – 70.2]). For punishment ratings (Figure S1c) there were main effects of number-of-shocks (*F*(2,48) = 11.03, p<0.001; partial
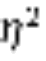
 = 0.315) and condition (*F*(2,48) = 14.2, p<0.001; partial
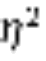
 = 0.372), indicating responders felt more inclined to punish the dictator when they received higher numbers of shocks, irrespective of their causation, and were overall more inclined to punish the dictator in the *Social-Intentioned* condition, where they believed the dictator to be responsible for the allocations. As expected, there was also a significant number-of-shocks x condition interaction (*F*(4,96) = 7.65, p<0.001; partial
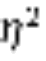
= 0.242), driven by a steeper effect of number of shocks in the *Social-Intentioned* condition, such that responders were most inclined to punish intentioned high allocations of shocks.

**Relationship between Experimental Data and Survey Measures**

Survey measures were compared against observed behavior with painful outcomes (mean offers made by dictators, and ratings of pain intensity, fairness, responsibility and inclination to punish by the responders) by means of linear regression. We found no significant relationships between any of the survey instruments used and either the mean offers made by dictators or the ratings made by responders.

**Supporting Figure 1**

**
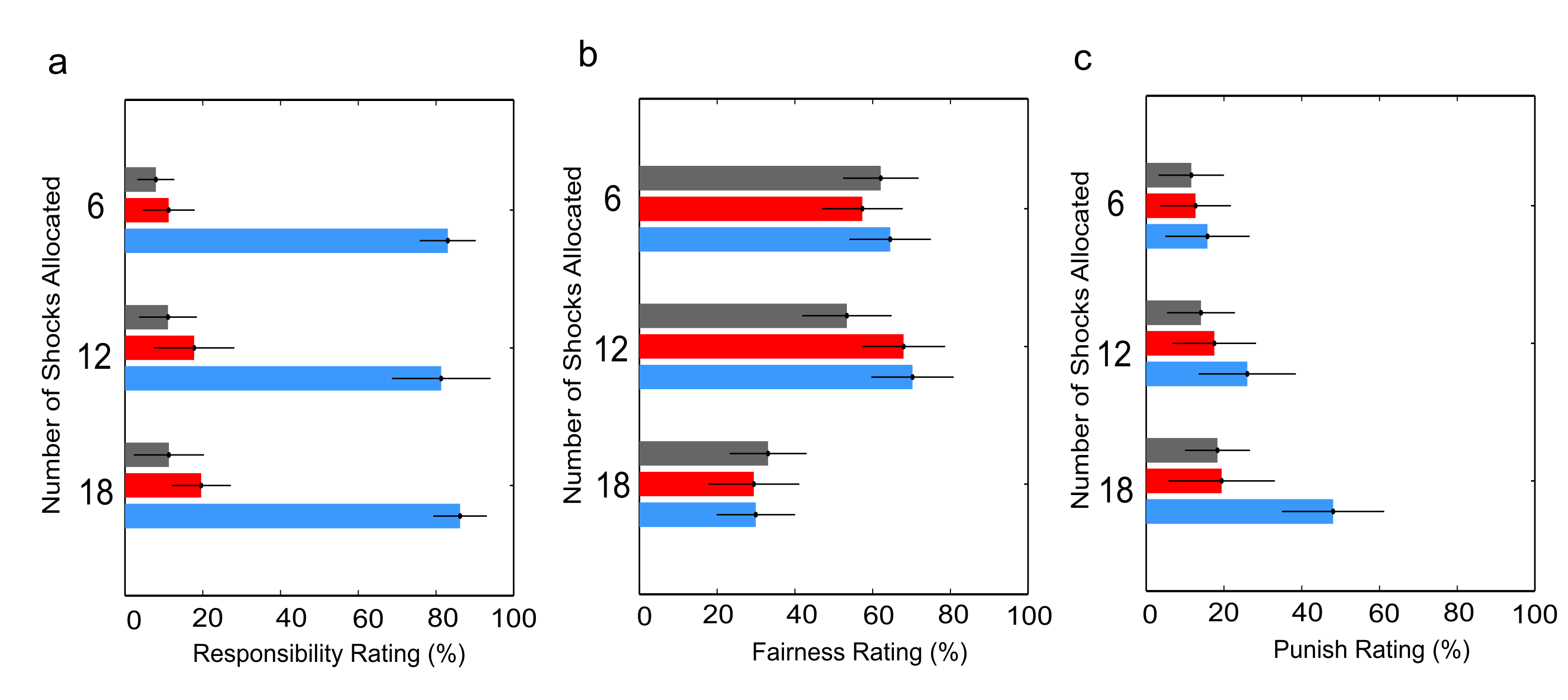
**

**Figure S1. Social Rating Scores Made by Responders.** Ratings made by responders (*N*=25) are plotted on the horizontal axes for the three conditions, Non-Social (uppermost, gray bar in each grouping), Social-Chance (middle, red bar) and Social-Intentioned (lowermost, blue bar), are grouped by number of shocks received (vertical axis category). Error bars represent 95% confidence intervals around the means, based on a Student’s *t*-distribution. **a** Responsibility ratings are highest in the Social-Intentioned condition, indicating a high degree of credibility in the experimental manipulation. **b** Fairness ratings. As expected participants rated receiving 18 shocks as less fair than receiving either 12 or 6 shocks, whilst the latter two outcomes were rated as equally fair when collapsing across conditions (estimated marginal mean rating for 18 shocks = 30.8%, 95% CI [21.5-40.2], 12 shocks = 63.9% [54.2-73.6], 6 shocks = 61.3% [52.5 – 70.2]). **c** Punishment ratings were consistent with the inclination to punish increasing with the number of shocks allocated by the dictator. There was a significant number-of-shocks x condition interaction (F(4,96) = 7.65, p<0.001), driven by a steeper effect of number of shocks in the Social-Intentioned condition, such that responders were most inclined to punish intentioned high allocations of shocks.
